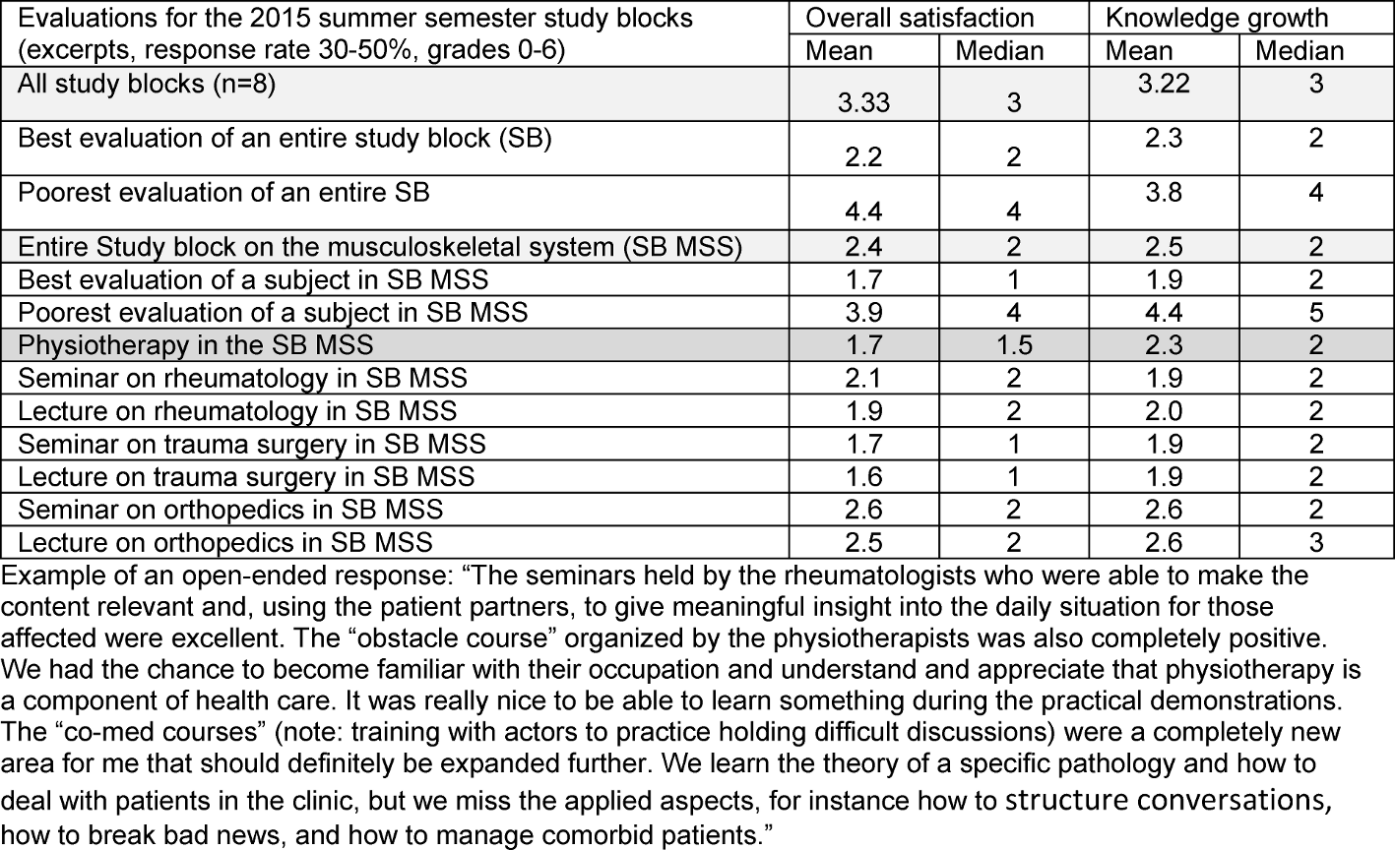# Interprofessional education as part of becoming a doctor or physiotherapist in a competency-based curriculum

**DOI:** 10.3205/zma001014

**Published:** 2016-04-29

**Authors:** Oliver Sander, Regine Schmidt, Gerd Rehkämper, Tim Lögters, Christoph Zilkens, Matthias Schneider

**Affiliations:** 1 Heinrich-Heine-Universität Duesseldorf, Poliklinik, Funktionsbereich & Hiller Forschungszentrum für Rheumatologie, Düsseldorf, Germany; 2 Universitätsklinikum Düsseldorf Ausbildungszentrum, Fachbereich Physiotherapie, Düsseldorf, Germany; 3 Heinrich-Heine-Universität Düsseldorf, Institut für Anatomie I, Düsseldorf, Germany; 4 Heinrich-Heine-Universität Düsseldorf, Klinik für Unfall und Handchirurgie, Düsseldorf, Germany; 5 Heinrich-Heine-Universität Düsseldorf, Orthopädische Klinik, Düsseldorf, Germany; 6 Heinrich-Heine-Universität Duesseldorf, Funktionsbereich & Hiller Forschungszentrum für Rheumatologie, Düsseldorf, Germany; 7 Heinrich-Heine-Universität Duesseldorf, Medizinische Fakultät, Studiendekanat, Düsseldorf, Germany

**Keywords:** interprofessional learning, physiotherapy, diseases of the musculoskeletal system, patient partner

## Abstract

**Introduction: **Interprofessional learning is a critical pre-requisite for future interprofessional work. Structural adaptations in education offer possibilities to introduce new concepts. Rheumatic and musculoskeletal diseases (RMD) are both prevented and treated by physicians and physiotherapists but the development of interprofessional roles is seldom part of curricula.

**Project description:** A complex, longitudinal interprofessional educational approach for future doctors and physiotherapists was designed and implanted at various stages (anatomy, physical examination, pathology, therapy). Most segments of the RMD curriculum are now based on interprofessional classes. Student satisfaction with learning is continually and comparatively evaluated. Learning success is assessed with practical and written exams.

**Results:** Interprofessional teaching was first introduced in 2013 for 420 first-year and 360 fourth-year medical students, along with 40 first- and third-year physiotherapy majors. The satisfaction with teaching and learning is high and distinctly above average for all teaching areas (satisfaction RMD rated as 2.4; average for all is 3.3). The percentage of those who pass the final exam is 94%. 100% of the students surveyed support the continuation of this interprofessional unit.

**Conclusion:** Interprofessional teaching of RMD can be successfully implemented for future physicians and physiotherapists at different learning levels.

## Zusammenfassung

**Einleitung: **Interprofessionelles Lernen ist sinnvolle Voraussetzung eines zukünftigen interprofessionellen Handelns. Strukturelle Änderungen in der Ausbildung bieten die Möglichkeit, neue Konzepte einzuführen. Rheumatische und muskuloskelettale Erkrankungen (RMD) werden von Medizinern und Physiotherapeuten vorgebeugt und behandelt.

**Projektbeschreibung: **Ein komplexes, longitudinales interprofessionelles Lehr- und Lernkonzept für angehende Mediziner und Physiotherapeuten wurde für alle Qualifikationsstufen der Ausbildung (Anatomie, körperliche Untersuchung, Krankheitslehre, Therapie) entwickelt. Große Abschnitte der Lehre zu RMD werden gemeinsam bestritten. Die Lernzufriedenheit wird kontinuierlich und vergleichend evaluiert. Der Lernerfolg wird in praktischen Prüfungen und Klausuren überprüft.

**Ergebnis: **Die interprofessionelle Lehre wurde vor 2 Jahren für 420 Studierende im 1. Studienjahr und 360 Studierende im 4. Studienjahr sowie 40 angehende Physiotherapeuten im 1. und 3. Ausbildungsjahr eingeführt und bis jetzt fortgesetzt. Die dokumentierte Lehr- und Lernzufriedenheit ist hoch und liegt deutlich über dem Durchschnitt aller Lehrbereiche (Zufriedenheit RMD Note 2,4, Durchschnitt aller 3,3) . Die Bestehensquote der Abschlussprüfungen liegt bei 94%. Alle Befragten befürworten die Fortsetzung der interprofessionellen Lehre.

**Schlussfolgerung: **Interprofessionelle Lehre zu RMD kann für angehende Mediziner und Physiotherapeuten in verschiedenen Qualifikationsstufen erfolgreich umgesetzt werden.

## 1. Introduction

Health professionals collaborate interprofessionally on a daily basis when providing health care to patients. This workplace reality is at present unaccounted by the educational programs in the different health care disciplines taught at German educational institutions and universities, where there is a conspicuous lack of interprofessional education. Based on international experiences [1], calls to develop approaches to interprofessional education in Germany are growing . An extensive position paper, complete with examples and comparisons of the approaches presently pursued in Europe and recommendations for implementation in the German-speaking countries, has been recently published in this journal [2] and already discussed and debated at the conference of the German Medical Association (*Bundesärztekammer)* [3], [http://www.aerzteblatt.de/nachrichten/62214/Interprofessionelles-Lernen-soll-zu-einer-besseren-Versorgung-fuehren]. Detected barriers are organizational, such as separate and autonomous responsibility for education in each of the different health professions, the educational approach with the more practice-oriented colleges of applied sciences on one and the academic study of medicine based on several years of pre-requisite theoretical learning on the other side.

Successful interprofessional education must be based on development of an educational concept, with all of the affected professions participating in this process. Long-established routines and reservations about interprofessional education must be overcome in order to implement successful projects [1], [4]. With the introduction of a competency-based curriculum in 2013 at the medical department of Heinrich Heine University in Duesseldorf, Germany, there was an opportunity to integrate interprofessional education as part of a complete restructuring of the educational program.

Rheumatic and musculoskeletal diseases (RMD) pose a great challenge for routine health care. They are not only the most common reason for prescribing medications, formally excusing patients from work, and even for early retirement, but also clearly limit quality of life and ability to participate [5], [6]. RMD often have multiple causes and need various therapies, many times simultaneously. Despite this knowledge, university teaching in Germany concerning this area is unsatisfactory, and the known deficits have yet to be remedied [7], [8].

The musculoskeletal system offers students a good place to start formal learning due to the relative ease of clinically examination, the wide experience of many with injuries, and the often simple, mechanical explanations. Physiotherapists and physicians play both important roles in the treatment of RMD, even though different approaches and therapies are generally available to them. Physiotherapy is often viewed during medical education as a less important and not evidence based form of therapy. Physiotherapists often view the physician primarily as the source of medical orders, and communication is usually limited to a few words or medical indication codes. Whoever has been fortunate enough to experience an interprofessional medical round or case consultation knows that joint assessment and cooperative therapy planning are distinctly more effective.

People who have RMD or who wish to prevent it are not just to be viewed as passive recipients of a therapy, but rather to be recognized as active participants who can prevent and treat through their behavior. Even patients must be trained and can be integrated into educational curricula.

RMD has many different causes: the basic ones are congenital disorders, age, injury and inflammation. Along with bones and joints, tendons and ligaments, muscles, fascia, nerves, vessels, skin, metabolism and the immune system are all necessary to maintain mobility. Even self-perception and the personal motivation to move and exercise appropriately are central factors in maintaining vitality.

Competency-based education regarding RMD is valuable and meaningful when done in an interdisciplinary, interprofessional manner in close cooperation with patients. In cooperation with the School of Physiotherapy and the patient self-help groups at the University Hospital, the Medical School at the Heinrich Heine University has created an opportunity for precisely this by developing and implementing a joint concept for teaching and learning.

## 2. Project description

The changes to the medical program were radical. The pre-clinical and clinical phases have been replaced by qualification phases; competencies and overarching learning objectives were formulated. The curriculum is now no longer sequenced according to subjects, but rather organized under interdisciplinary topic headings.

In the following we present how RMD has been included in the curriculum in terms of form and content with a focus on interprofessional education.

The teaching of RMD was developed by Rheumatology together with Trauma Surgery and Orthopedics at the different qualification levels; the RMD unit is now jointly taught by these three departments. The design of the educational approach took place over four years with equal participation of all involved subject areas independent of profession. Suggestions were reviewed by the university’s teaching commission and included in the curriculum. The instructors and student representatives meet regularly, at least three times during the academic year, to further optimize teaching, address uncertainties and strengthen interprofessionalism. A coordinator is available to help with short-term solutions.

The School of Physiotherapy has restructured its curriculum so that a group of students from one cohort are able to participate in the interprofessional education unit, while the remaining students continue with their practical training.

The interprofessional education unit began with the implementation of the competency-based curriculum in Düsseldorf during the 2013/14 winter semester. A total of 420 medical students in their first year of study, 360 fourth-year medical students and 80 future physiotherapists (40 in the first year of training and another 40 in the third) have now participated in this joint learning program. Continuation is intended: in the 2016/17 winter semester the students who began in 2013 will have reached the second qualification phase.

### 2.1. First qualification phase

**Figure 1:**
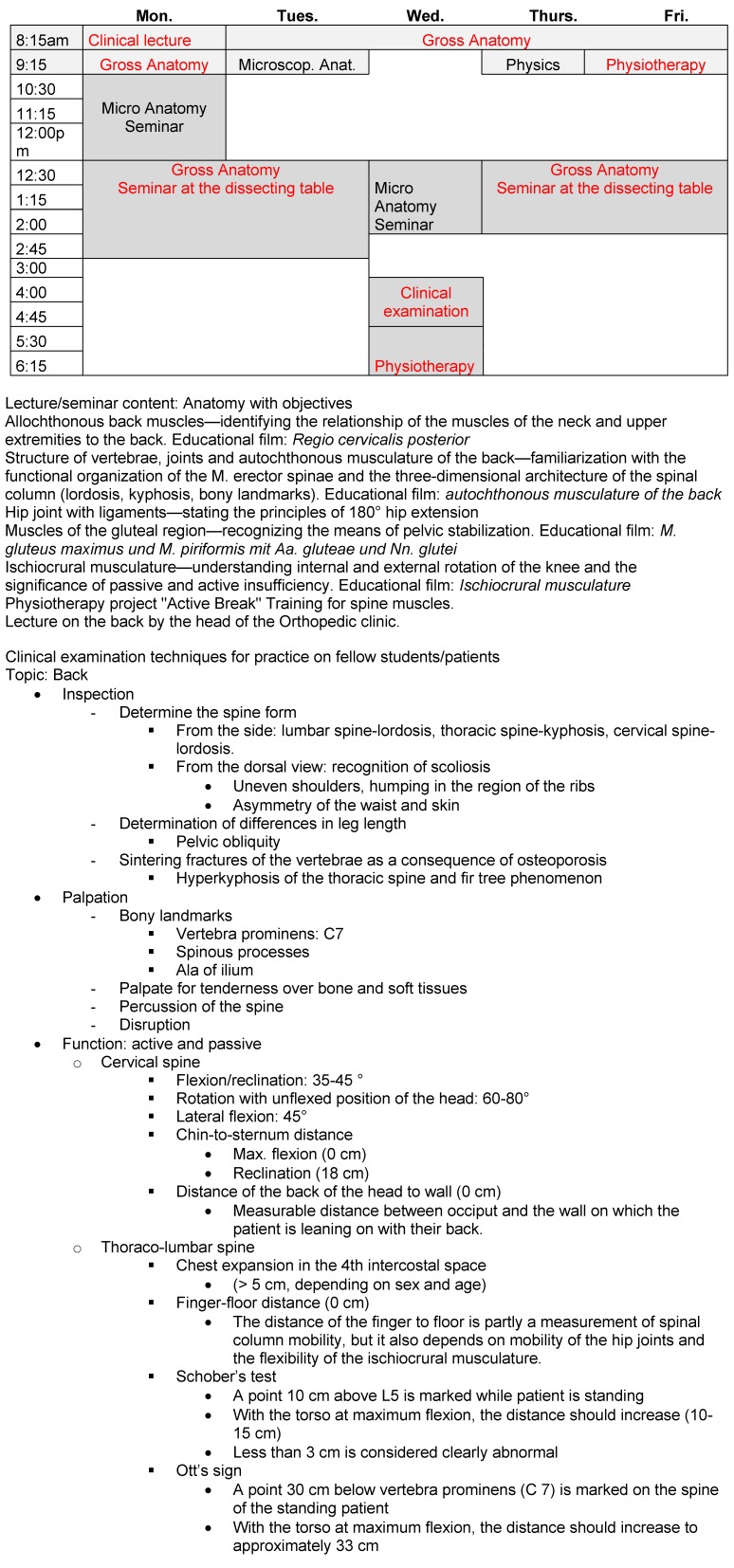
Example given for a topic: Course schedule for the 11th week of the first semester; Seminar group topic: “The back”, Lectures 8:15-10:30 am, followed by a seminar or practical instruction, interprofessionally designed instruction is indicated in red

Examining the healthy musculoskeletal system is taught in the first year of study. In a block focusing on the human body and movement, gross anatomy is covered theoretically and practically by the anatomical institutes (dissection course). As this is accomplished, basic physical terms are elucidated in an integrated manner and, in close cooperation with the School of Physiotherapy, physical therapy concepts are presented to students early on in the form of practical exercises. Shoulder, hand, knee, back, and foot are the focal topics that are each covered in an interdisciplinary and interprofessional manner over the period of a week (see figure 1 (Fig. 1)). The content of the practical component is the recognition of what is normal, its variability and range, as well as the first deviations, meaning deformations of the spine, pelvic obliquity, etc. Orthopedists and trauma surgeons explain common and typical injuries based on physical principles allowing a better understanding of anatomy. At the same time, concepts regarding the preservation of a healthy musculoskeletal system are presented (exercises to strengthen the back or knees, etc.). For this, it was possible to involve experienced physiotherapists and well-known coaches from a local German athletic training center (see figure 1 (Fig. 1)).

Tutors (advanced physiotherapy [PT] students and medical students with a higher qualification) present, explain and practice examination techniques on humans with the first-year students. Physical therapy is presented and learned at work stations. Future physiotherapists have the chance to participate in gross anatomy.

**Figure 2:**
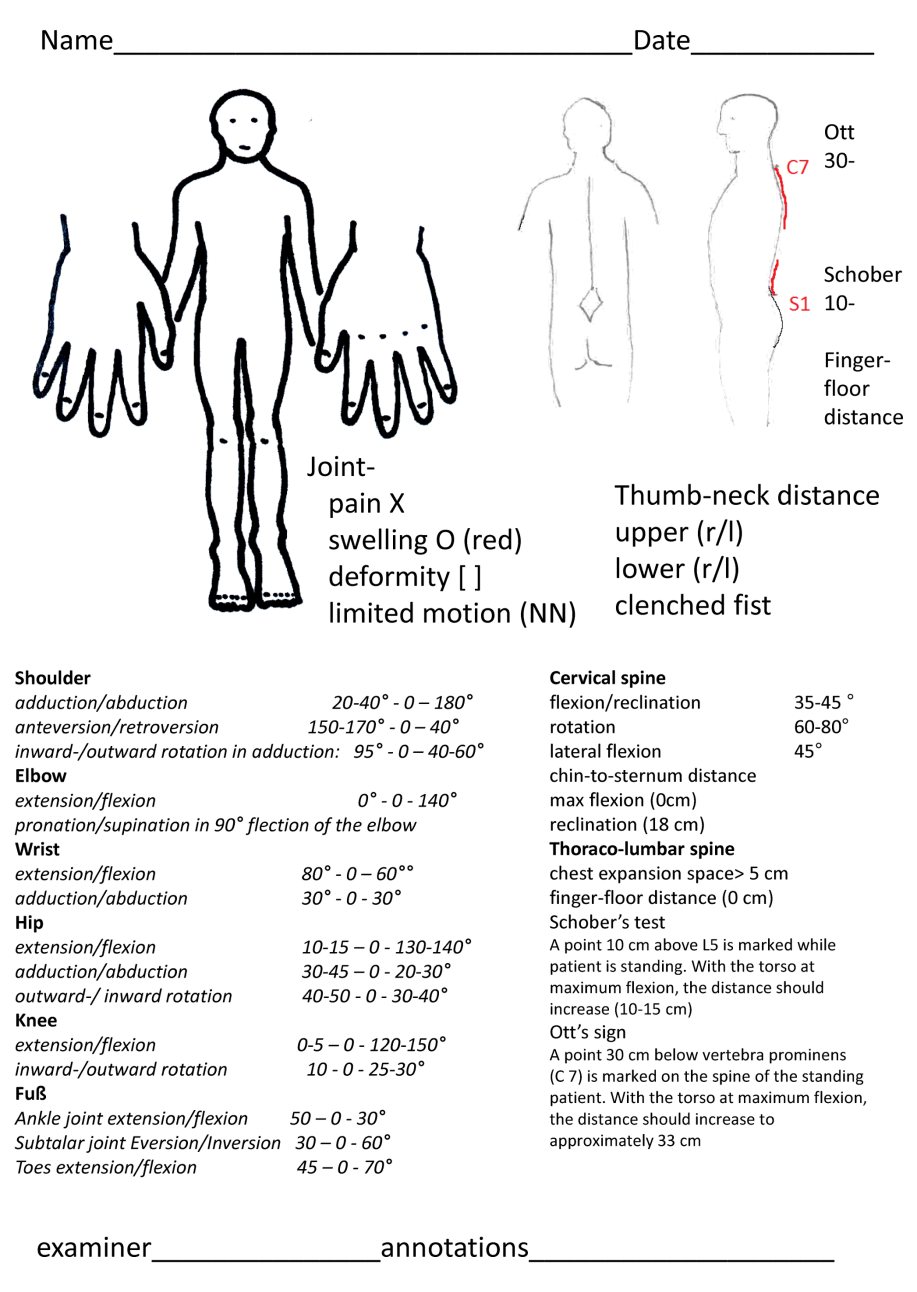
Standardized sheet for the internship in general practice

During the second semester, general practitioners teach how to record medical case histories. Patients in the local ankylosing spondylitis self-help group participate as example patients on whom students can practice. Future medical doctors can then apply this knowledge of recording case histories and examining the musculoskeletal system during the first general practice internship. A standardized method of documentation was developed for this purpose (see figure 2 (Fig. 2)). The participating general practitioners have taken part in a training session on examination techniques held by the student tutors.

The future physiotherapists participate in the regularly scheduled rehab sports sessions held by the self-help groups and gather experience in leading exercise groups. Having interested medical students participate in the rehab sports program as part of an elective curriculum is intended.

During the third year of study, medical students receive more detailed instruction on clinical diagnostics, differential diagnostics, medical measures and immunology (infection and defense). Since this is only of partial relevance to the topic of RMD and of no relevance for future physiotherapists, PT students were not included.

#### 2.2. Second qualification phase

Starting in the fourth year of study, multiple four-week practical blocks are offered with instruction involving patients (outpatient care or operating room), of which there is one with RMD-specific subjects. These blocks have already been presented in this journal [9]. Just like the medical students, the PT students observe outpatient care for those with rheumatism.

The second qualification phase for RMD is structured by a four-week block dealing with the musculoskeletal system. Third-year PT students take equal part in the entire study block. They also take the same final multiple-choice exam. This 60-hour unit is divided into 24 hours of lecture (for 90 medical students and 10 PT students) and 36 hours of seminar (in groups of 15 medical students and 2 PT students), and one week of individual study of cases (CASUS) and preparation for the exam.

**Figure 3:**
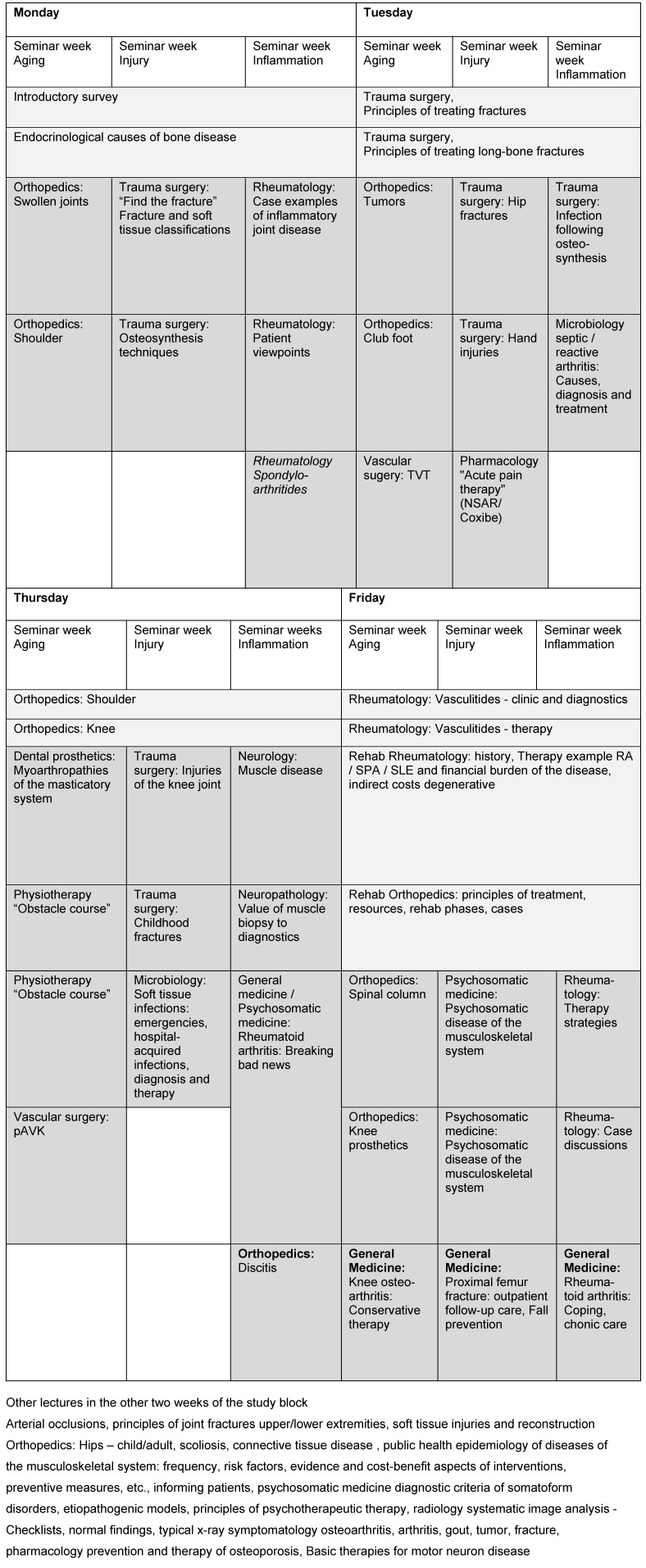
Example course schedule for a week during the fourth year, lectures 8:15-10:00 am, Fridays until 12:00 pm (light gray), all else as seminars (dark) or practical instruction. Each represents one hour of instruction, all interprofessional, with no classes taking place on Wednesdays.

Major weekly topics cover the musculoskeletal system and aging, accidents and inflammation. Instruction is based on cases that are initially covered in small groups at the start of the week. Along with the core subjects, important aspects are taught in seminars on human genetics, neuropathology, microbiology, psychosomatics, epidemiology, etc., and at the end of each week a summary from the perspective of general practice is presented (see figure 3 (Fig. 3)).

During this study block, the future physiotherapists also offer a two-hour seminar as a type of “obstacle course.” Trained patients who are members of the Rheuma-Liga, a self-help league, stand in to enable special examinations, connections with daily life, and information on dealing with disease and finding helpful resources, etc. [https://www.rheuma-liga.de/aktivitaeten/projekte/detailansicht/news/patient-als-partner/]. Using actors as patients allows stressful situations where communication needs to take place to be practiced and contemplated.

#### 2.3. Third qualification phase

Medical students meet up again with the future physiotherapists on hospital wards during their practical year (6^th^ year of study) and have the opportunity to jointly provide patient care in an inpatient setting. Both groups of students also serve as tutors for the subsequent semesters.

#### 2.4. Evaluation

This curricular unit is regularly evaluated by the students, and the results analyzed by the office of the Dean of Studies. Due to organizational reasons, it was not possible to have intervention and control groups dealing with the same topic.

In addition, the students participating in the second round during the 2015 summer semester were surveyed anonymously regarding their expectations and experiences with interprofessional education, as well as their opinion regarding the future of this educational approach.

Pass rates for questions addressing RMD on the central exams could serve over the medium term to evaluate the learning success of the future physicians.

The enthusiasm of future physicians for the area of RMD could be measured using applicant numbers at the pertinent advanced professional training institutes.

For rheumatology, there is longitudinal, forward-looking and regional data generated at the patient level in the form of the core documentation compiled by the cooperating rheumatism centers for use and implementation of different therapies, such as physiotherapy for rheumatism patients, function status, participation and patient satisfaction [http://dgrh.de/fileadmin/media/Forschung/Versorgungsforschung/ErwachsenenKerndok/Standardpraesentation_2013_extern.pdf]. This could allow for recognition of healthcare-related regional trends and serve long-term validation at the care level.

## 3. Results

### 3.1. Implementation from the perspective of medical education

The model presented here takes the main focus from teaching individual subjects and places it on the patients and the health care team. It creates acceptance for different points of view and demonstrates their benefit and usefulness. In addition to content, important skills and competencies are experienced and thus made real. The affected patient, physiotherapist and physician learn together and from each other.

Gross anatomy instructors are seeing that the functional understanding of the musculoskeletal system in the dissection course profits immensely from experiencing the anatomy of living bodies (physical examination course, physiotherapy).

All of the instructors who took part in the survey reported positive experiences and supported the continuation of this approach (n=7, <10%).

#### 3.2. Implementation from the perspective of the medical students

Future medical doctors approached this opportunity to experience interprofessional education with great curiosity, but had no particular expectations (response rate to voluntary survey n=12, 13%). Individual students expressed concern that, as a result of the course, there would be less time available to learn the material covered by the multiple-choice exams.

The first-semester students were at first more uncertain about performing physical exams on each other than were the third-year students who had already received instruction in examination techniques. This was addressed right away by including an explanatory introduction. The willingness to participate in the physical examination was more visible among the future physiotherapists and the medical students took this as a “cue” to do the same. 

##### 3.4.1. Student satisfaction

Student satisfaction with this curricular unit as measured in the central teaching evaluation (n=37, 41% response rate), knowledge gain and satisfaction with course structure and sequence were with a mean rating of 2.4 distinctly better than the evaluation results for the other topic-specific study blocks, which received mean ratings of 3.3 (response rate 30-50%, see figure 3 (Fig. 3)). Teaching done by physiotherapists was evaluated particularly positively by the medical students with a rating of 1.7. The practical learning is evaluated higher in the practical block that includes the RMD clinics, something that allows the assumption of an additional effect on student perception of their own practical team skills and competencies.

In terms of general satisfaction and learning progress, structured teaching using patients and Physiotherapists is evaluated by medical students as being equal to instruction by medical doctors (see figure 4 (Fig. 4)).

##### 3.4.2. Passing rates

The learning objectives assessed by the centralized final exams (multiple-choice) are achieved by the great majority of the students. The passing rate for medical students was 94% with a minimum passing grade of 60%; a total of 14% earned the grade of very good, 58% that of good, 14% satisfactory, and 8% achieved sufficient results.

A measurable effect of interprofessional education as it is described here on the passing rate for the centralized exams has not yet been ascertained.

#### 3.3. Implementation from the perspective of the physiotherapists

Access to the university’s electronic learning materials that accompanied the course was not possible at first for the PT students; however, it was possible to arrange for special access.

The future physiotherapists experienced the shared learning environment with the medical students as a successful meeting of the two professional groups, who should be closely interlinked for the purpose of providing optimal patient care. Assuming the role of tutor early on, physiotherapy students were able to practice asserting confidence when giving patient care. The future physiotherapists experienced a high level of professional esteem as tutors and the open curiosity of the medical students. The written responses of the medical students regarding the expertise and motivated friendliness of the physiotherapists also obviously provided impetus for even closer collaboration on the wards during the internship. During the subsequent internships, almost all participants continued the productive interaction with those whom they met during the RMD study block. News of the existence of such constructive cooperation has spread and, in turn, led to further synergetic effects in providing therapy and negotiating therapy goals with patients in concrete situations. Physiotherapists realize that they are not only able to but even should contribute to giving physicians deeper insights into the possibilities of physiotherapy so that these therapies can be prescribed in a more targeted manner. Arriving at therapy goals in a team comprised of all those involved offers a basis for the best possible health care. Through joint learning in the module on the musculoskeletal system, a forum for collaboration on equal footing is implicitly experienced and becoming as self-evident as the goal of providing the best possible health care. On the standardized evaluations, all of the respondents (n=4, 30%) were satisfied with the interprofessional education.

Of the future physiotherapists, 91.7% passed the final exam on which there was a minimum passing grade of 50%.

## 4. Discussion

While it is easier to implement interprofessional education at medical schools at which training for different professions is offered, as is common in other countries [10], many differing educational forms must be coordinated to accomplish this in Germany. If there is a willingness to change and commit, this can be done on the instructor level. Test dates, school breaks, access to internet sites, all of these pose surprisingly obstinate barriers to implementing interprofessional courses. This can quickly lead to failure if decision-makers have even the smallest doubt regarding the endeavor [4]. In addition, the administrative effort is disproportionately high for small projects making it a barrier that explains the lack of widespread implementation.

We are able to present a concept that has been implemented successfully focusing on interprofessional learning between patients, medical and physiotherapy students. Our approach fulfills not only the minimum requirements set by the position paper of the GMA committee on Interprofessional Education for the Health Professions [2], but is also primarily designed longitudinally for the entire educational program/study during the different qualification phases and influences an entire subject area (musculoskeletal system).

The practical implementation and subsequent evaluation are simpler for the program presented here than for small isolated solutions, for instance those affecting only one seminar. Interprofessional education has become a matter of course in our case.

When planning and implementing the curricular changes here, we were able to count on the support of faculty members for program restructuring and a practical focus on competency, as well as on an enthusiastic team representing various professions as being a great help.

Individual sequences, such as the physiotherapy “obstacle course,” had already been tried out in other settings (Rheumatology Summer School). As a result, the risk of failure could be reduced.

However, the success of such an extensive project is certainly not foreseeable and can only be ensured by frequent re-evaluations. To accomplish this, standardized and required (anonymous) evaluations are helpful, as long as the response rate is assured. The voluntary evaluation was in this case thoroughly supportive and positive, but is not representative with a response rate among instructors of 10%. At the student level, evaluations are for the most part established and can be used as a helpful measure. Organized participation of PT students in the educational design and development has not yet been implemented. This is crucial for long-term success.

Confirming the educational approach through passing rates on the central exams is the next step to be taken in this project.

When designing an interprofessional approach to teaching and learning, attention should be paid to the steps described by Hall and Zierler [4]; sufficiently motivated colleagues and sufficient time are also necessary. A direct relevance of this kind of comprehensive interprofessional education to other professions, such as nursing or medical technology, seems not to be possible.Although there are separate aspects that do overlap such as care giving, imaging, and lab diagnostics, the depth of learning in diagnostics and pathology is distinctly more generalized than it is in medical education.

## Competing interests

The authors declare that they have no competing interests.

## Figures and Tables

**Figure 4:**